# Supplementary material for: The Systems Biology Research Tool: evolvable open-source software
Source: BMC Syst Biol. 2008 Jun 29;2:55. doi: 10.1186/1752-0509-2-55 (PMC2446383; doi:10.1186/1752-0509-2-55)
Supplement: Additional file 1 — SBRT Archive. An archive of the current version of the Systems Biology Research Tool. [file 1752-0509-2-55-S1.zip › sbrt-1.4.0/doc/developers_guide/api/sbrt/kernel/math/stat/comp/class-use/CorrResult.html]

Uses of Interface sbrt.kernel.math.stat.comp.CorrResult


|  |  |  |
| --- | --- | --- |
| **Overview** \| **Package** \| **Class** \| **Use** \| **Tree** \| **Deprecated** \| **Index** \| **Help** | |  |
| PREV   NEXT | **FRAMES**    **NO FRAMES**     **All Classes** |  |


---


## **Uses of Interface sbrt.kernel.math.stat.comp.CorrResult**

| Packages that use CorrResult | |
| --- | --- |
| **sbrt.ext.R.stat** | Provides classes that allow communication with R for the purposes of statistics. |
| **sbrt.kernel.math.stat.comp** | Provides classes and interfaces for performing statistical computations. |
| **sbrt.kernel.math.stat.proc** | Provides classes and interfaces for performing statistical processes. |

Uses of CorrResult in sbrt.ext.R.stat

| Classes in sbrt.ext.R.stat that implement CorrResult | |
| --- | --- |
| `static class` | `RCorrelator.Result`             This class is used to store the results of computing a correlation using R. |

Uses of CorrResult in sbrt.kernel.math.stat.comp

| Classes in sbrt.kernel.math.stat.comp with type parameters of type CorrResult | |
| --- | --- |
| `interface` | `Correlator<V,T extends CorrResult>`             This interface is used to represent correlation statistic computers. |

| Classes in sbrt.kernel.math.stat.comp that implement CorrResult | |
| --- | --- |
| `class` | `TauResult`             This class is used to store the results of a correlation computation. |

Uses of CorrResult in sbrt.kernel.math.stat.proc

| Classes in sbrt.kernel.math.stat.proc with type parameters of type CorrResult | |
| --- | --- |
| `class` | `CorrelationAnalysis<V,T extends CorrResult>`             This class is used to perform correlation experiments. |
| `class` | `MultipleCorrelationAnalysis<V,T extends CorrResult>`             This class is used to perform multiple correlation analyses. |

---


|  |  |  |
| --- | --- | --- |
| **Overview** \| **Package** \| **Class** \| **Use** \| **Tree** \| **Deprecated** \| **Index** \| **Help** | |  |
| PREV   NEXT | **FRAMES**    **NO FRAMES**     **All Classes** |  |


---
